# From lessons on the long‐term effects of the preimplantation environment on later health to a “modified ART‐DOHaD” animal model

**DOI:** 10.1002/rmb2.12469

**Published:** 2022-06-29

**Authors:** Md Wasim Bari, Shiori Ishiyama, Sachi Matsumoto, Kazuki Mochizuki, Satoshi Kishigami

**Affiliations:** ^1^ Department of Integrated Applied Life Science University of Yamanashi Yamanashi Japan; ^2^ Faculty of Life and Environmental Sciences University of Yamanashi Yamanashi Japan; ^3^ Center for advanced Assisted Reproductive Technologies University of Yamanashi Yamanashi Japan

**Keywords:** ART, DOHaD, MEM mouse. preimplantation, type 2 diabetes

## Abstract

**Background:**

At its earliest stages, mammalian embryonic development is apparently simple but vulnerable. The environment during the preimplantation period, which only lasts a couple of days, has been implicated in adult health, extending to such early stages the concept of the developmental origin of health and disease (DOHaD).

**Methods:**

In this review, we first provide a brief history of assisted reproductive technology (ART) focusing on in vitro culture and its outcomes during subsequent development mainly in mice and humans. Further, we introduce the “MEM mouse,” a novel type 2 diabetes mouse model generated by in vitro culture of preimplantation embryos in alpha minimum essential medium (αMEM).

**Main findings:**

The association between ART and its long‐term effects has been carefully examined for its application in human infertility treatment. The “MEM mouse” develops steatohepatitis and kidney disease with diabetes into adulthood.

**Conclusion:**

The close association between the environment of preimplantation and health in postnatal life is being clarified. The approach by which severe mouse phenotypes are successfully induced by manipulating the environment of preimplantation embryos could provide new chronic disease animal models, which we call “modified ART‐DOHaD” animal models. This will also offer insights into the mechanisms underlying their long‐term effects.

## Graphical abstract

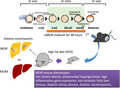

## 1 INTRODUCTION

**FIGURE 1:**
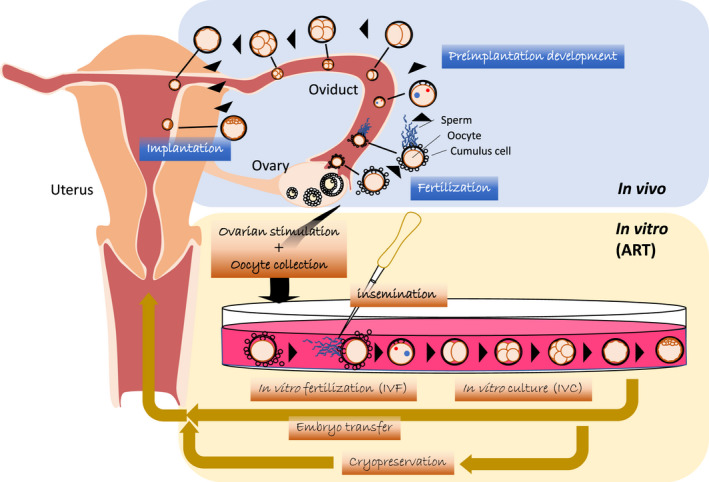
Schematic flow illustrating the human in vivo and in vitro fertilization (ART). In ART, embryos experience different environments in vitro

The successful initiation of mammalian embryonic development occurs when the parental gametes unequally cooperate to share their genetic materials. A haploid sperm cell gives a haploid oocyte its properties, including the genomic information (DNA sequence) with its epigenetic marks,^1^ which activate the oocyte during fertilization.^2^ On the other hand, in addition to the maternal genome, the oocyte provides its cytoplasm comprising a variety of factors, the so‐called maternal factors, which are required for the subsequent development of the fertilized embryo.^3^ In mammals, fertilization normally takes place within the ampullary region of the fallopian tube, followed by preimplantation development (Figure 1). The success in generating offspring after in vitro fertilization (IVF), in vitro culture (IVC), and embryo transfer (ET) in mammals in the early to mid‐20th century, allowed to treat several cases of infertility in humans.^4^ Over 8 million IVF babies have been born in the world since the birth of the first IVF baby, Louise Brown, reported in 1978 by Robert Edwards and colleagues.^4^ In the last decade, a number of new approaches besides IVF have been developed and integrated into routine assisted reproductive technology (ART) practices, including blastocyst stage ET, cryopreservation of embryos, and preimplantation genetic screening.^5^ Although these approaches are considered beneficial for infertility treatment, vulnerable embryos unexpectedly have to experience in vitro environments that differ from those encountered in vivo (Figure 1). The embryonic exposure to different environmental factors such as nutrition may lead to long‐term consequences including altered growth and phenotype characteristics.^6^

Epidemiology has studied the long‐term effects of the environment in early life on the future health of individuals since the early 20th century.^7^ For example, in 1934, an association between childhood conditions and later mortality was suggested from death rates for England and Wales since 1845, and for Sweden since 1751.^8^ Cohort studies including one on Dutch famine near the end of World War II (1944–1945), further revealed how extreme nutritional environments can affect fetal development and future health, leading to schizophrenia, depression, coronary heart disease, type 2 diabetes, among other disease conditions.^9^, ^10^ These studies suggest that the effects of the environment depend on their timing during gestation, with early gestation being the most vulnerable period.^9^ In the last decades, these associations have been refined through various studies in a variety of research fields including clinical, epidemiological, and animal experimental research, resulting in the concept of developmental origins of health and disease (DOHaD).^11^, ^12^ According to this DOHaD concept, “the risk of developing some chronic non‐communicable diseases in adulthood is influenced not only by genetic and adult lifestyle factors but also by environmental factors acting in early life.” Further, this association is expanded to refer not only to environmental exposures taking place in early life but also before life, such as those affecting the parents.^13^ Thus, the concept can provide a universal platform to study the associations between environmental factors at any stage of life and the outcomes on future health.

The DOHaD concept is applicable not only to in vivo environmental factors such as the nutrition status of pregnant mothers but also to the in vitro environment of embryos notably during preimplantation, which leads to concerns regarding the effect of ART on embryos’ future health.^6^ In this review, we first focus on the outcomes of IVC on subsequent development and phenotypes mainly in the mouse. Second, we introduce a new unique type 2 diabetes model mouse, the “MEM mouse,” which presents complications that include steatohepatitis, glomerulosclerosis, and arteriolosclerosis in the kidney as diabetic kidney disease (DKD), simply by exposure to alpha minimum essential medium (αMEM) for 48 h from the two‐cell embryo stage.^14^, ^15^, ^16^

## 2 EFFECTS OF IN VITRO CULTURE ON FUTURE HEALTH

### 2.1 Effect of in vitro culture media on preimplantation development

In the mid‐20th century, Whitten succeeded in culturing mouse embryos from the eight‐cell to the blastocyst stage using a modified Krebs–Ringer‐bicarbonate medium with glucose and egg white.^17^ McLaren and Biggers reported a live birth after transferring embryos to the recipient uteri even after in vitro culture.^18^ In 1959, Chang first succeeded in obtaining a live birth by rabbit IVF^19^ following the finding of sperm capacitation.^20^, ^21^ About 10 years later, mouse IVF was successfully achieved.^22^ Thus, over half a century has passed since the early success of in vitro embryo culture in mammals, during which, culture media have been much improved.^23^, ^24^ Two major approaches allowed to optimize their chemical composition and concentration: “*back*‐*to*‐*nature*” which aims to mimic human oviduct and uterine fluids in the female reproductive tract, resulting in the human tubal fluid medium,^25^ and “*let the embryos choose*” which aims to maximize the developmental rate and notably yielded the KSOM medium.^26^ However, even these well‐developed media are not optimal and cause stress to the embryos compared to the in vivo situation.^27^ Preimplantation embryos must adapt to their cultural environment to survive and, consequently in vitro culture itself impacts not only on their intrinsic developmental genetic program and viability but also on their future health.^6^, ^13^

### 2.2 Impact of IVF/IVC on subsequent development and health

The numerous studies using human ART and animal models suggest that preimplantation embryos are highly vulnerable and sensitive to environmental conditions that can affect their future growth and health.^6^, ^13^ For example, poor maternal nutrition even exclusively during preimplantation development results in adult excess growth and hypertension especially in female mouse offspring.^28^ After IVF compared to natural mating, the mouse offspring weigh more at birth, while females show delayed glucose clearance with more insulin secretion.^29^ Therefore, human ART raised concerns in terms of increasing the risk of developing type 2 diabetes and cardiovascular diseases in adults, although more studies are needed to reach strong conclusions.^30^ How can the environmental conditions of preimplantation embryos contribute, in a couple of days, to increasing such disease risk in the future? There are several good models including maternal low protein diet and IVF which allow dissecting this association. Here, we focus on the differences between IVF/IVC and in vivo embryos to provide such insights.

**FIGURE 2:**
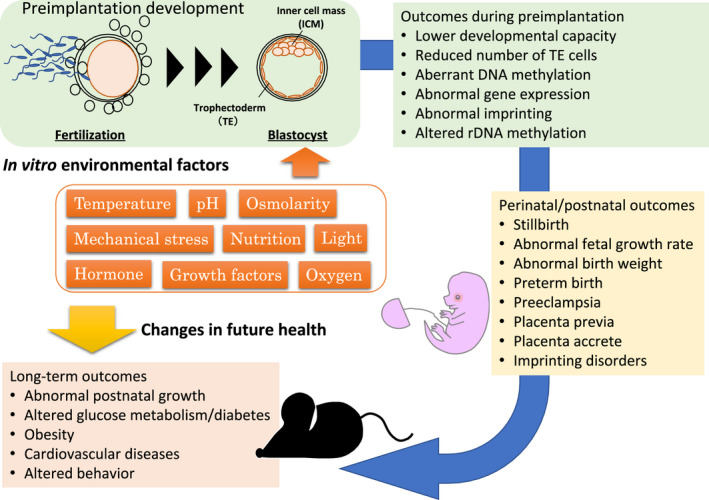
Schematic flow illustrating the embryo's possible short‐term and long‐term outcomes after ART

Based on animal model studies, IVF/IVC reduces during preimplantation the number of trophectoderm (TE) cells, which give rise to tissues in the placenta, but also increases cell death of the blastocysts^31^, ^32^, ^33^ and alters their global gene expression,^31^, ^34^ compared to in vivo fertilized embryos (Figure 2). The altered genes in the IVF embryos are notably related to apoptosis, cell differentiation, metabolism, and protein synthesis.^31^, ^33^ To overcome these adverse consequences of IVC during the preimplantation period, co‐culture systems with oviduct epithelial cells, supplementation with oviductal fluid, or with extracellular vesicles (EVs) have been reported to mimic in vivo conditions.^32^ Among the findings, supplementation with EVs derived from the oviduct increased the birth rates after ET in mice, with decreased apoptosis and improved cellular differentiation of the embryos.^35^

### 2.3 Impact of IVF/IVC on the embryonic epigenome

In addition to disturbing gene expression in the IVF/IVC embryos, epigenetic alterations caused by ART have been intensively studied, revealing disturbance and fixation on their genomes in the long term.^36^, ^37^, ^38^ In particular, as an epigenetic modification, DNA methylation, referring to the attachment of a methyl group to cytosine, plays a crucial role in the regulation of genome functions including gene expression, genomic imprinting, and X‐chromosome inactivation during embryonic development and cell differentiation, considering its stable inheritance and dynamic changes as a cellular memory system.^39^ It is thus expected that disturbance in the epigenome occurs under IVF/IVC since environmental conditions such as the diet can modify the epigenetic state of the genome,^40^, ^41^ widely considered as the nutrigenomics.^42^ For example, after fertilization, dynamic modifications of the epigenome involving DNA methylation further occur during preimplantation development.^43^, ^44^, ^45^ One‐carbon metabolism (OCM) implicates the methionine/folate cycles to provide 1C units (methyl groups) for protein synthesis, DNA synthesis, and redox control.^46^ This OCM also provides S‐adenosylmethionine, which is the methyl donor for most methyltransferases, allowing to addition of DNA, RNA, lipids, and histone, among others. Increased dietary intake of folic acid which provides OCM supply during the periconceptional period can increase DNA methylation of the *IGF2* gene in the DNA of human offspring.^47^ Conversely, restricting as folic acid and methionine from the periconceptional diet of mature female sheep leads to the exposed offspring to an alteration of DNA methylation in the fetal liver, together with heavier body weight, elevated blood pressure, and insulin resistance to adulthood.^48^

After fertilization, epigenetic reprogramming occurs, with allelic differences within a cell, derived from the distinct epigenetic profiles of the sperm and oocyte on their genomes.^36^, ^37^, ^38^ Genomic imprinting, which affects a subset of genes in mammals to generate a monoallelic, parental‐specific expression pattern, depends on DNA methylation.^49^ In human ART studies, the correlation between ART and increased incidences of imprinting disorders such as Beckwith–Wiedemann syndrome (BWS) has been reported.^37^, ^50^ This syndrome is associated with aberrant methylation patterns at the imprinting control regions (ICRs) of *IGF2*/*H19* and *CDKN1C*/*KCNQ1OT1*.^51^ For example, the incidences of BWS were increased 4.46‐fold higher in ART compared to naturally conceived children, which may take place during IVF or ICSI and IVC.^52^ Similarly, aberrant methylation patterns at the *Igf2*/*H19* ICR were observed in IVF mouse models.^53^ Furthermore, aberrant DNA methylation patterns caused by ART were partially rescued by maternal intake of moderate folic acid supplementation in mouse embryos and placenta.^54^ Thus, environmental conditions along ART processes including IVC could deeply impact the DNA methylation status in the conceptus.

Setting abnormalities of imprinting genes aside, the targets of aberrant DNA methylation causing long‐term consequences after ART‐assisted birth have remained largely unknown. Abnormal regulation of ribosomal DNA (rDNA), of which transcription is a limiting step in ribosome biogenesis for protein synthesis, is proposed to underlie this association between abnormal DNA methylation status and long‐term consequences considering findings using a mouse low protein diet (LPD) model. When LPD was provided only during the preimplantation period, rDNA methylation was increased in the preimplantation embryos, which decreased rRNA expression and conversely caused abnormal excess of rDNA transcription during adult life, affecting cell growth and fate determination, and increasing the risk of adult cardiometabolic disease.^55^ Investigating how rDNA transcription is affected in embryos and adults after IVF or IVC is thus considered important.^56^ While further studies identifying the genes causing the long‐term effects of IVF/IVC are warranted, DNA methylation is assumed to represent one of the important changes leading to adverse developmental programming.

### 2.4 Perinatal and long‐term outcomes associated with IVF/IVC

Although most IVF children are healthy, accumulating evidence suggest increased risks of outcomes associated with IVF, such as stillbirth, fetal growth restriction, low birth weight, preterm birth, preeclampsia, placenta previa/accreta, increased growth trajectory in infancy, as well as metabolic and cardiovascular defects in later life, in addition to imprinting disorders as mentioned above (Figure 2).^57^, ^58^, ^59^, ^60^, ^61^ An association between birth weight and later chronic diseases including cardiovascular diseases has been suggested from epidemiological observations, contributing to the DOHaD concept.^62^, ^63^ Therefore, in both IVF‐ and spontaneously conceived children, it is important to identify the causal mechanism underlying altered prenatal development in terms of outcomes on future health. How can we dissect causal relationships following IVF/IVC?

The theory of “placenta‐derived diseases”^64^ provides key insights and a comprehensive understanding of the abnormalities induced by ART, including IVF/IVC.^65^ The placenta forms an interface between the fetus and its mother to sustain fetal development by providing the mother with all the nutrients and oxygen, functioning as a barrier against maternal hormones and immune system as well as parasites, and acting as an endocrine organ.^66^, ^67^ The theory of “placenta‐derived diseases” stipulates that “if normal placenta is impaired or the organ's capacity for adaptation exceeded, then the fetal milieu may be perturbed with major consequences for the life‐long health of the offspring,”^65^ based on accumulating evidence of strong associations between placental phenotypes and chronic diseases, following the DOHaD concept.^68^

Accumulating evidence suggest that ART increases the risk of abnormal placental phenotypes such as placenta previa, greater placental weight, placental metabolic alterations, and abnormal gene expression.^65^ Consistently, in mouse, ART treatments reduce fetal weight and induce placental overgrowth at embryonic day 18.5, resulting in defects of placental layer segregation and glycogen cell migration.^69^ These ART treatments also downregulate placental nutrient transporters and reduce placental efficiency.^69^ The ART placentae exhibit increased methylation levels at ICRs of *H19* with abnormal expression of imprinted genes which are important for placental development and function.^69^ Another recent mouse study dissected the effect of distinct ART approaches such as hormone stimulation, IVF, IVC, and ET, which revealed that IVC itself causes placental overgrowth, as well as reduces fetal weight and placental DNA methylation, while placental expression levels of sFLT1, an anti‐angiogenic protein, increase after IVF/IVC as increased circulating maternal levels of sFLT1 are implicated in causing maternal symptoms of preeclampsia in humans.^70^ Therefore, among the ART procedures, IVC is considered one of the most critical factors causing placental abnormalities that disturb placental function and lead to chronic diseases.

### 2.5 “MEM mouse” as a “modified ART‐DOHaD” animal model

Preimplantation embryos re‐establish their developmental program and trajectory depending on their environment, at least partly in contexts of abnormal placental functions caused by altered DNA methylation patterns during IVC. However, the precise underlying mechanisms remain largely unknown to address many questions. For instance, which particular stage of preimplantation development is critical for changing the programming? How much time is necessary for rewriting the program? What environmental factors can change the program? How many target genes or signal pathways are involved in generating the phenotypes? How many different phenotypes are programable? In addressing these questions, and others, various animal models are expected to provide valuable insights. First, it is important to study animals presenting mutations involved in the regulation of placental development, in particular those causing intrauterine growth restriction and pre‐eclampsia,^71^ as well as mutant animals with type 2 diabetes mellitus and obese phenotypes such as *ob*/*ob* mice.^72^ Second, it is important to investigate various “ART‐DOHaD” animal models, produced by ART and shown to exhibit long‐term effects,^73^ integrated into “DOHaD” animal models produced by maternal nutritional imbalance such as under‐ and overnutrition.^74^ Since ART in domestic animals including cattle, sheep, and horses is worldwide used, pre‐ and peri‐natal effects have been studied to resolve ART‐associated problems such as low pregnancy rates, prolonged gestation, and fetal overgrowth, also known as the large offspring syndrome (LOS).^73^ As a result of studying causative factors, for example, for LOS in cattle, which presents as an aberrant development of the placenta,^75^ the inclusion of serum in embryo culture medium and co‐culture with oviductal cells were identified mainly to cause abnormal feto‐placental development in ruminants.^73^, ^76^

**FIGURE 3:**
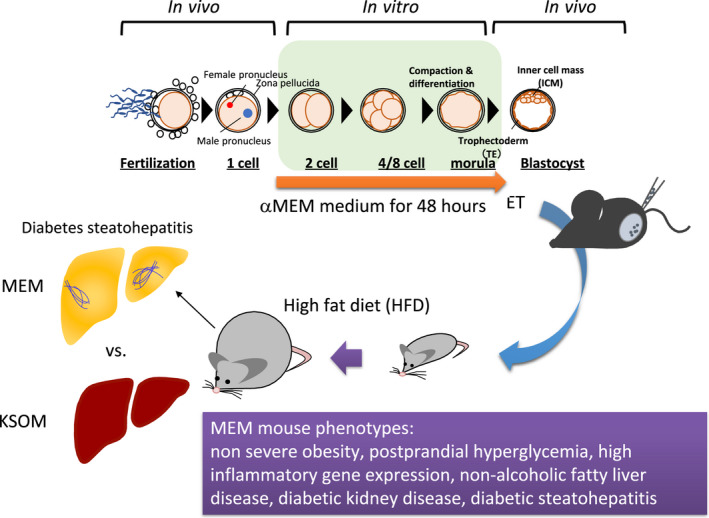
Schematic flow of the MEM mouse as a “modified ART‐DOHaD” animal model

Finally, it appears critical to study different types of “modified ART‐DOHaD” animal models with more severe phenotypes and a higher penetrance upon embryo exposure to synthetic microenvironmental factors such as nutritional and chemical stressors, to decode the rewritten programs. For example, treatment of zygotes with trichostatin A, an inhibitor of histone deacetylase, for 24 h after fertilization was shown to induce epigenetic changes that include hyperacetylation, resulting in reduced birth weight in IVF offspring contrary to offspring derived from the somatic‐cell nuclear transfer. This finding provides an example of a long‐term effect caused by the chemical alteration of epigenetic modifications during preimplantation.^77^, ^78^, ^79^ Recently, we have reported that mice derived from embryos treated with just αMEM medium, which is commonly used for mammalian cell culture, over 48 h, so‐called the “MEM mouse” exhibit increased weight with severe type 2 diabetes‐related phenotypes such as postprandial hyperglycemia, high inflammation gene expression, non‐alcoholic fatty liver disease, DKD, diabetic steatohepatitis, which are not observed in mice derived from embryos cultured in KSOM‐AA (mKSOM) medium, a KSOM medium supplemented with amino acids (Figure 3).^26^, ^80^ These results imply that the “MEM mouse” can be used as a novel animal model for human diabetes.^14^, ^15^, ^16^ Although the underlying mechanisms induced by αMEM medium remain unknown, unlike KSOM‐AA, this αMEM medium has no protein such as bovine serum albumin (BSA) while containing vitamins such as folic acid (Table 1). Considering that BSA in culture media plays an important role beyond a source of amino acids,^81^ its absence may contribute to inducing the phenotype. It should be noted that the osmolarity of KSOM‐AA and αMEM media are also sort of different such as around 270 and 285–315 mOsm/kg, respectively.^80^, ^82^ Future studies with the MEM mouse will focus on detailing the phenotype and identifying causal factors, and required conditions for induction of the MEM mouse phenotype such as the timing and duration of αMEM medium exposure, characterizing its placental abnormality, and elucidating its epigenetic alterations to address the above questions.

**TABLE 1:** Compositions of culture media for preimplantation embryos

	Components (mg/L)	KSOM‐AA^26^, ^80^	αMEM^82^
Inorganic components	NaH_2_PO_4_	–	140
	KH_2_PO_4_	48	–
	CaCl_2_2H_2_O	251	265
	MgSO_4_7H_2_O	49	200
	NaCl	5552	6800
	KCl	186	400
	EDTA (2Na)	4	–
	NaHCO_3_	2100	2200
Organic components (amino acids, vitamins, others)	d‐glucose	36	1000
	Lactate‐Na	1132	–
	Pyruvate‐Na	22	110
	BSA	5000	–
	l‐Glutamine	150	292
	Amino acids	0.5×	1×
	Ascorbate‐Na	–	50
	d‐Biotin	–	0.1
	Choline‐Cl	–	1.0
	Folic acid	–	1.0
	i‐inositol	–	2.0
	Lipoic acid	–	1.0
	Niacinamide	–	1.0
	d‐1/2Ca Pantothenate	–	1.0
	Pyridoxal HCl	–	1.0
	Riboflavin	–	0.1
	Thiamine HCl	–	1.0
	Vitamin B_12_	–	1.4

## 3 CONCLUSION

Over the last two decades, our understanding pertaining to ART including IVC and its long‐term effects has much advanced based on the DOHaD concept. This DOHaD concept is also pertinent to other fields such as evolutionary developmental biology (evo‐devo) and ecological developmental biology (eco‐devo), which together provide a framework for understanding when and how environmental stressors modify the phenotypes of individuals, then result in chronic diseases over the life cycle through epigenetic regulation.^83^ Combined with protocols allowing to design “modified ART‐DOHaD” animal models with desired phenotypes by manipulating the microenvironment during preimplantation, studies of “modified ART‐DOHaD” animal models are expected to contribute not only to improving the culture medium for ART required to produce healthy offspring, to developing drugs and foods for the treatment of chronic diseases but also to decoding the underlying developmental programs. In this review, we do not cover all the work related to ART‐induced long‐term consequences considering that excellent Review papers have already been published^38^, ^65^ but instead, we insist on the importance of studying “modified ART‐DOHaD” animal models which are expected to contribute to elucidating the mechanisms underlying ART‐induced long‐term consequences on health.

## CONFLICT OF INTEREST

The authors declare no conflict of interest.

## 4 HUMAN AND ANIMAL RIGHTS

Non‐applicable for a Review article.
